# Pharmacological and Biochemical Characterization of TLQP-21 Activation of a Binding Site on CHO Cells

**DOI:** 10.3389/fphar.2017.00167

**Published:** 2017-03-30

**Authors:** Laura Molteni, Laura Rizzi, Elena Bresciani, Roberta Possenti, Pamela Petrocchi Passeri, Corrado Ghè, Giampiero Muccioli, Jean-Alain Fehrentz, Pascal Verdié, Jean Martinez, Robert J. Omeljaniuk, Giuseppe Biagini, Anna Binda, Ilaria Rivolta, Vittorio Locatelli, Antonio Torsello

**Affiliations:** ^1^Department of Medicine and Surgery, University of Milano-BicoccaMonza, Italy; ^2^PhD Program in Neuroscience, Department of Medicine and Surgery, University of Milano-BicoccaMonza, Italy; ^3^Department of Medicine of Systems, University of Rome “Tor Vergata”Rome, Italy; ^4^Department of Drug Science and Technology, University of TurinTurin, Italy; ^5^Centre National de la Recherche Scientifique, Max Mousseron Institute of Biomolecules UMR5247, University of Montpellier, École Nationale Supérieure de Chimie de MontpellierMontpellier, France; ^6^Department of Biology, Lakehead University, Thunder BayON, Canada; ^7^Laboratory of Experimental Epileptology, Department of Biomedical, Metabolic and Neural Sciences, University of Modena and Reggio EmiliaModena, Italy

**Keywords:** TLQP-21, VGF, calcium, SOCE, STIM-1, receptor, CHO, HFHH-10

## Abstract

VGF is a propeptide of 617 amino acids expressed throughout the central and the peripheral nervous system. VGF and peptides derived from its processing have been found in dense core vesicles and are released from neuronal and neuroendocrine cells via the regulated secretory pathway. Among VGF-derived neuropeptides, TLQP-21 (VGF^556-576^) has raised a huge interest and is one of most studied. TLQP-21 is a multifunctional neuropeptide involved in the control of several physiological functions, potentially including energy homeostasis, pain modulation, stress responsiveness and reproduction. Although little information is available about its receptor and the intracellular mechanisms mediating its biological effects, recent reports suggest that TLQP-21 may bind to the complement receptors C3aR1 and/or gC1qR. The first aim of this study was to ascertain the existence and nature of TLQP-21 binding sites in CHO cells. Secondly, we endeavored to characterize the ligand binding to these sites by using a small panel of VGF-derived peptides. And finally, we investigated the influence of TLQP-21 on selected intracellular signaling pathways. We report that CHO cells express a single class of saturable and specific binding sites for TLQP-21 with an affinity and capacity of *K*_d_ = 0.55 ± 0.05 × 10^-9^ M and *B*_max =_ 81.7 ± 3.9 fmol/mg protein, respectively. Among the many bioactive products derived from the C-terminal region of VGF that we tested, TLQP-21 was the most potent in stimulating intracellular calcium mobilization in CHO cells; this effect is primarily due to its C-terminal fragment (HFHH-10). TLQP-21 induced rapid and transient dephosphorylation of phospholipase Cγ1 and phospholipase A2. Generation of IP_3_ and diacylglycerol was crucial for TLQP-21 bioactivity. In conclusion, our results suggest that the receptor stimulated by TLQP-21 belongs to the family of the G_q_-coupled receptors, and its activation first increases membrane-lipid derived second messengers which thereby induce the mobilization of Ca^2+^ from the endoplasmic reticulum followed by a slower store-operated Ca^2+^ entry from outside the cell.

## Introduction

The *vgf* gene, originally identified as a *nerve growth factor* responsive gene in PC12 cells (Levi et al., 1985), has a tissue-specific pattern of expression limited to specific neurons and to specific endocrine cells (Salton et al., 2000; Levi et al., 2004). In rodents, the *vgf* gene encodes a 617 amino acid protein which is included in the extended granin family and is named secretogranin VII (Bartolomucci et al., 2006). Secretogranin VII itself is proteolytically processed to yield more than ten different bioactive peptides (Trani et al., 2002). In the rat brain, VGF is expressed in areas involved in the regulation of feeding, reproduction, stress responsiveness and general homeostasis (Salton et al., 2000; Levi et al., 2004; Razzoli et al., 2012), and VGF-derived peptides have been found significantly decreased in some neurodegenerative diseases (Carrette et al., 2003; Ruetschi et al., 2005; Cocco et al., 2010). VGF immunoreactivity was, as well, reported in gonadotroph and lactotroph cells in the rat anterior pituitary (Ferri et al., 1995).

**GRAPHICAL ABSTRACT:**
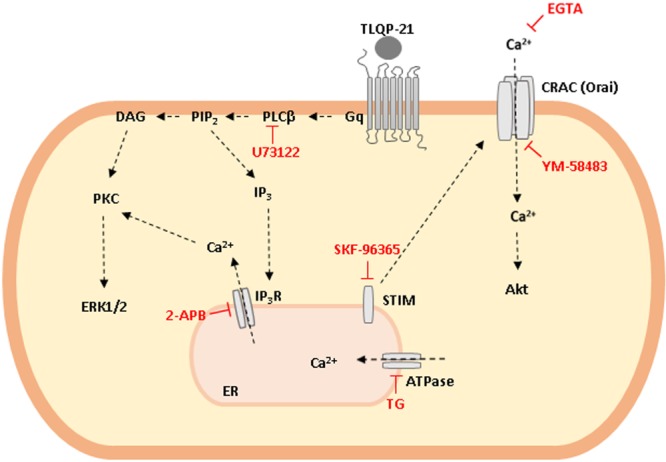
**Schematic representation of TLQP-21 intracellular transduction mechanism in CHO cells.** TLQP-21, by binding a G protein coupled receptor (GPCR), activates PLCβ that in turn produces DAG and IP_3_ as second messengers. These molecules activate PKC, stimulate ERK1/2 phosphorylation and induce intracellular Ca^2+^ release from the ER with the subsequent Ca^2+^ entry from outside the cell. Phosphorylation of AKT is probably a result of the increase in cellular Ca^2+^ concentrations.

Among several bioactive peptides derived from VGF, TLQP-21 (VGF^556-576^) is a 21 amino acid peptide which has been immunopurified from brain tissues (Bartolomucci et al., 2006). Despite many efforts to characterize the physiological effects of TLQP-21, little is known about its molecular targets. Nonetheless, adipocytes express a high affinity binding site for TLQP-21 and in these cells TLQP-21 stimulates a pro-lipolytic effect (Possenti et al., 2012). Moreover, we have recently demonstrated TLQP-21 binding sites on CHO cells through the use of Atomic Force Microscopy (Cassina et al., 2013). Interestingly, the rat ovary express high levels of TLQP-21, which has been proposed to affect female reproduction by modulating pituitary LH release (Aguilar et al., 2013; Noli et al., 2014) The statistical distribution of the attractive force between TLQP-21 and its binding site is indicative of a single class of binding sites. This presence of a TLQP-21 binding site in these cells is consistent with their unique dose- and time-dependent increases of intracellular calcium (Ca^2+^) mobilization in response to TLQP-21 (Cassina et al., 2013). TLQP-21 stimulation of intracellular Ca^2+^ was concentration-dependent, whereas LRPS-21 (a scrambled control peptide that contains the same amino acids residues of TLQP-21 but rearranged in a random order to prevent homology with other published proteins) did not stimulate Ca^2+^ increases in CHO cells, confirming the specificity of TLQP-21 effects. Furthermore, N38 cells, which do not respond to TLQP-21, do not express binding sites for TLQP-21 in the Atomic Force Microscopy measurements (Cassina et al., 2013).

It has recently been proposed that the complement component 3a receptor 1 (C3aR1) mediates TLQP-21 signaling in CHO cells, and that TLQP-21 could be a natural agonist of this receptor (Hannedouche et al., 2013). Noteworthy, it has been reported that the stimulation of CHO cells with TLQP-21 did not induce any measurable intracellular Ca^2+^ increase unless cells were subjected to a strong priming with 100 μM ATP (Hannedouche et al., 2013). Other authors have also reported that in rat macrophages TLQP-21 binds specifically to the complement component C1q receptor (gC1qR) (Chen et al., 2013). Interestingly, gC1qR and C3aR1 are receptors for complement protein and it is possible that TLQP-21 interacts with both receptors; however, which receptor mediates the effects of TLQP-21 is still object of debate.

Since TLQP-21 is emerging as a novel target for obesity-associated disorders (Bartolomucci et al., 2006; Possenti et al., 2012), diabetes (Stephens et al., 2012), neuropathic pain (Chen et al., 2013; Fairbanks et al., 2014) and other human pathologies (Cocco et al., 2010), the purpose of this study was to better characterize the binding site for TLQP-21 in CHO cells, investigating its binding characteristics and the intracellular pathways activated by the peptide-receptor interaction. The data presented here clearly indicate that TLQP-21 binds to a single class of receptors, probably belonging to the family of the G_q_-coupled receptors, and stimulates intracellular Ca^2+^ release primarily from the endoplasmic reticulum (ER).

## Materials and Methods

### Chemicals

TLQP-21 (TLQPPASSRRRHFHHALPPAR), YATL-23 (YATLQ PPASSRRHFHHALPPAR), TLQP-62 (TLQPPASSRRRHFHHA LPPARHHPDLEAQARRAQEEADAEERRLQEQEELEN-YIEH VLLHRP), TLQP-8 (TLQPPASS), HFHH-10 (HFHHALPP AR), HHPD-41 (HHPDLEAQARRAQEEADAEERRLQEQEELE NYIEHVLLHRP), and LRPS-21 (LRPSHTRPAHQSFARP LHRPA) have been synthesized by us using conventional solid phase synthesis.

Cyclosporine A (CsA), thapsigargin (TG), U73122, 2-Aminoethyl diphenylborinate (2-APB), SKF-96365, YM-58483, and EGTA were purchased from Sigma–Aldrich (St Louis, MO, USA). Unless otherwise specified, all other reagents were from Sigma–Aldrich.

### Cell Cultures

CHO cells were cultured in HAM’S F12 medium supplemented with 10% heat-inactivated foetal bovine serum (FBS), 100 IU/ml penicillin, 100 μg/ml streptomycin, and 2 mM L-glutamine (all Euroclone, Pero, Italy) under standard cell culture conditions (at 37°C, in 5% CO_2_).

### Intracellular Ca^2+^ Mobilization Assay

CHO cells were plated at 20.000 cells/well into black walled, clear bottom 96-well plate (Corning, Germany) and cultured one day up to 80–90% of confluence. Prior to assay, cells were incubated in dark conditions with 100 μl of Hank’s Balanced Salt Solution (HBSS) containing 20 mM HEPES, 2.5 mM probenecid and 4.5 μM FLUO-4 NW (Molecular Probes, Eugene, OR, USA) at 37°C and 5% CO_2_ for 45 min. Fluorescence emissions were measured with the multilabel spectrophotometer VICTOR^3^ (Perkin Elmer, MA, USA) at 485/535 nm (excitation/emission filters) every 0.5 s for the 20 s preceding and the 60 s following the stimulation. TLQP-21, TLQP-62, TLQP-8, HHPD-41, HFHH-10, and LRPS-21 were diluted in HBSS solution and injected into the wells by an automated injector system. The nature of Ca^2+^ stores involved in TLQP-21 action was investigated preincubating cells with TG (2 μM, 20 min), an inhibitor of the Ca^2+^-ATPase pump responsible for sequestering Ca^2+^ in the ER (Feng et al., 2008), or CsA (2 μM, 15 min), an inhibitor of the mitochondrial permeability transition pore (PTP) (Liantonio et al., 2007). To ascertain whether phospholipase C (PLC) and inositol trisphosphate receptors (IP_3_R) were involved in TLQP-21 mechanism of action, cells were incubated with a specific PLC inhibitor (U73122, 10 μM for 10 min) and a IP_3_R antagonist (2-APB, 75 μM for 15 min) before the injection of TLQP-21 (Macmillan and McCarron, 2010; Li et al., 2013). Depletion of Ca^2+^ from the ER leads to Ca^2+^ entry from outside the cell by activation of Store-Operated Channels (SOCs). Again, to assess the involvement of this pathway, CHO cells were incubated with selective antagonists of this pathway (SKF-96365 and YM-58483 10 μM for 20 min, EGTA 1 mM for 30 min) before the injection of TLQP-21 (Ishikawa et al., 2003; Li et al., 2013).

### TLQP-21 Binding Assay

Binding of TLQP-21 to crude membranes (30,000 × *g* pellet) obtained from CHO cells was carried out using [^125^I]-YATL-23 as ligand. YATL-23 was radioiodinated (specific activity, 2000 Ci/mmol) using the lactoperoxidase method (Muccioli et al., 1998; Muccioli et al., 2001) by Perkin Elmer (Milan, Italy) and purified by reverse phase high-performance liquid chromatography. For the single point binding assay, cell membranes (corresponding to 100 μg membrane protein) were incubated in triplicate at 23°C, unless otherwise noted, for 4 h under constant shaking with 0.5 nM [^125^I]-YATL-23 in a final volume of 0.5 ml assay buffer (50 mM Tris, 2.5 mM EGTA, 0.002% bacitracin, 0.1% bovine serum albumin, titrated to a final pH of 7.4 with HCl). Non-specific binding was measured in parallel incubations with 1 μM unlabelled YATL-23 to displace the radioligand. Similar results were obtained using TLQP-21 to displace the radioligand. The binding reaction was terminated by the addition of ice-cold assay buffer, followed by rapid filtration through Whatman GF/B filters as previously reported (Muccioli et al., 1998) and the radioactivity bound to membranes was measured by a Packard auto-γ-counter. Specific binding-values were calculated as the difference obtained subtracting non-specific from total binding. Specific binding was expressed as a percentage of the total radioactivity added. For saturation binding studies, cell membranes were incubated with various concentrations of the radioligand (0.03–4 nM). Competition studies were performed by incubating cell membranes (150 μg/tube) with 1 nM [^125^I]-YATL-23 with or without various concentrations (from 10 pM to 0.1 μM) of unlabelled YATL-23, TLQP-21, or LRPS-21. Data were plotted and curves fit using the GraphPad Prism software version 4 (GraphPad Software, San Diego, CA, USA) assuming that the binding was due to a single class of binding sites, thus allowing determination of the maximum binding capacity (B_max_), dissociation constant (K_d_), Hill slope and concentration of the competitor causing 50% inhibition (IC_50_) of specific radioligand binding.

### Phosphorylation of Cellular Kinases

CHO cells were plated 24 h before time course experiments in 35 mm dishes at 70% confluence. After three washes with medium w/o serum, cells were serum-starved for 1 h. Time course experiments were performed and after quick removal of the medium the reaction was stopped by placing the dish on ice and adding 100 μl of ice-cold lysis buffer (50 mM Tris-HCl, pH 7.5, 150 mM NaCl, 10 mM EDTA) containing a protease inhibitor cocktail and PhosphoStop inhibitor cocktail (Roche Diagnostic, Mannheim, Germany).

Cells were stored at –80°C until further processing. Cells were harvested and equivalent amounts of cell extracts (corresponding to approximately 200.000 cells) were run on NuPAGE precast 4–12% gradient gels (Invitrogen, USA) and transferred to a polyvinylidene difluoride (PVDF) membrane (Amersham). After staining with Ponceau S to verify uniformity of protein load/transfer, membranes were analyzed for immunoreactivity. Incubation with primary antibodies (anti-phospho-AMPK (Thr172); anti-phospho-AKT (Ser473); anti-phospho-ERK1/2 (Thr202/Tyr204); anti-phospho-PKC (Ser660); anti-phospho-PLA2 (Ser505); anti-phospho-JNK (Ser63); and anti-phospho-PLCγ1 (Tyr783) rabbit polyclonal antibodies; Cell Signaling Technology, Danvers, MA, USA) at 1:1.000 dilution was performed overnight at 4°C (Petrocchi et al., 2010). Incubation with peroxidase-coupled secondary antibodies (Amersham, Arlington Heights, IL, USA; now GE; 1:5.000) was performed for 1 h at room temperature. Immunoreactivity was developed by enhanced chemiluminescence (ECL system; Amersham GE Healthcare, UK). Two parallel gels were run for each experiment and with probes for different anti-phosphorylated protein antibodies as indicated, avoiding stripping protocols. The gels were normalized with anti-β-actin monoclonal antibodies (Sigma). β-actin was chosen since we have previously observed that its levels remained stable in time, wherease those of α-tubulin decreased over time (Petrocchi et al., 2010).

### Statistical Analysis

Values are expressed as mean ± SEM. The statistical significance of differences between groups was evaluated with Tukey–Kramer’s t-test for multiple comparisons, preceded by the analysis of variance (ANOVA). Where appropriate, F-values and degrees of freedom (DF) are indicated in the legend of figures. A P-value of less than 0.05 was considered significant.

## Results

### TLQP-21 Stimulates Intracellular Ca^2+^ Levels in CHO Cells

**FIGURE 1:**
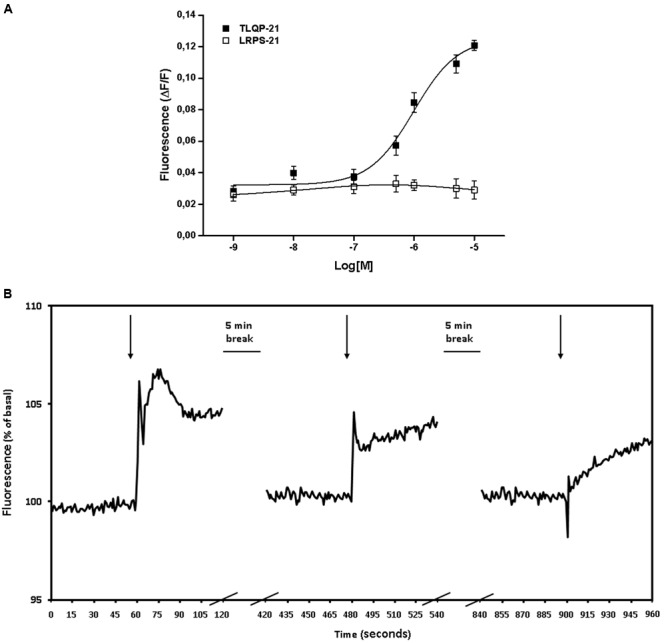
**TLQP-21 stimulation of intracellular Ca^2+^ levels in CHO cells. (A)** Cells were loaded with FLUO-4 NW and fluorescence emissions were measured at 485/535 nm (excitation/emission filters) every 0.5 s for the 60 s following injection of the stimuli. TLQP-21 was applied at concentrations ranging from 1 nM to 10 μM. Ca^2+^ mobilizing activity of TLQP-21 is plotted in terms of maximal response obtained at each given concentration in CHO cells. To ascertain the specificity of the effects, cells were also challenged with 1 nM–10 μM LRPS-21, a scrambled peptide containing the same amino acid residues of TLQP-21. **(B)** Effects of repeated stimulation with TLQP-21 on intracellular Ca^2+^ levels in CHO cells. 10 μM TLQP-21 was applied three times at 5 min intervals. Fluorescence was recorded every 0.5 s. Results are the means ± SEM of measurements obtained in at least six different wells for each experiment. All experiments have been repeated at least three times.

CHO cells were incubated *in vitro* with increasing concentrations (1 nM–10 μM) of TLQP-21. TLQP-21 (0.1 μM–10 μM) evoked acute and significant increases (*P* < 0.05) in intracellular Ca^2+^ levels in CHO cells (**Figure 1A**); by comparison, LRPS-21 tested at the concentration interval of 1 nM–10 μM did not stimulate Ca^2+^ levels in CHO cells (**Figure 1A**) thereby confirming the specificity of TLQP-21 action.

In the next series of experiments, we investigated whether CHO cells could respond to repeated TLQP-21 stimulations given at 5 min intervals from each other. Levels of free intracellular Ca^2+^ increased sharply upon the first challenge with 10 μM TLQP-21, whereas 5 min later a second application of 10 μM TLQP-21 induced only a blunted increase in cell fluorescence; no effects were observed when 5 min later 10 μM TLQP-21 was applied a third time (**Figure 1B**). A final challenge with 10 μM ATP to check for cell viability was not affected (data not shown).

### Calcium Mobilizing Effects of VGF-Derived Peptides

**FIGURE 2:**
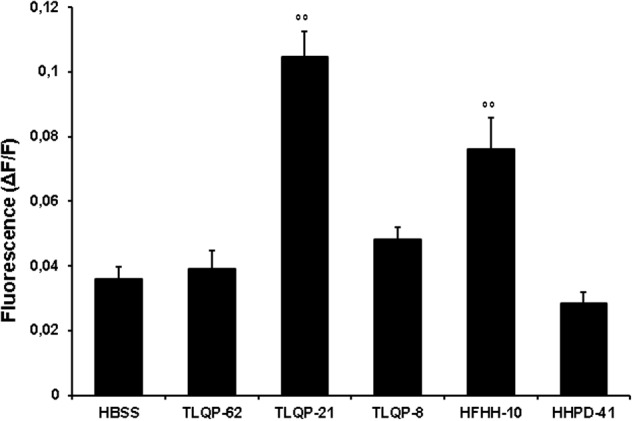
**VGF-derived peptides stimulation of intracellular Ca^2+^ levels in CHO cells.** Cells were loaded with FLUO-4 NW and stimulated with different VGF-derived peptides (1 μM). Ca^2+^ mobilizing activity of peptides is plotted in terms of maximal response obtained at each given concentration in CHO cells. Values plotted have been calculated as the difference between stimulated fluorescence and pre-stimulated fluorescence divided by pre-stimulated fluorescence (ΔF/F). Results are the means ± SEM of measurements obtained in at least five different wells for each experiment. ANOVA: *F* = 23.3; DF = 29. All experiments have been repeated at least three times. ^∘∘^*p* < 0.01 vs. HBSS

To gain further insight into the biological activity of VGF-derived peptides, we stimulated CHO cells with different peptides derived from the C-terminal region of VGF (**Table 1**). Natural processing of TLQP-62 by prohormone convertases yields TLQP-21 and HHPD-41 (**Table 1**). The 1 μM concentration has been chosen to compare the effects of TLQP-62 and its fragments on intracellular Ca^2+^ levels since this concentration was close to the EC50 of TLQP-21 and could have allowed to measure whether a test compound was less or more active than TLQP-21. At concentrations as large as 1 μM TLQP-62 failed to stimulate any significant increase in intracellular Ca^2+^ levels (**Figure 2**). Similarly, HHPD-41 was also ineffective, whereas TLQP-21 significantly stimulated intracellular calcium mobilization (**Figure 2**). TLQP-21 itself is a substrate for prohormone convertases to yield TLQP-8 (8 amino acids at the N-terminal of TLQP-21) and HFHH-10 (10 amino acids at the C-terminal of TLQP-21). HFHH-10 effectively stimulated an intracellular Ca^2+^ increase in CHO cells; by comparison, TLQP-8 at 1 μM induced only a slight and non-significant increase in intracellular Ca^2+^. These results strongly suggest that the C-terminal region of TLQP-21 is the sequence primarily involved in the stimulation of its receptor on CHO cells (**Figure 2**).

**Table 1:** Aminoacidic sequence of peptides of the C-terminal region of VGF.

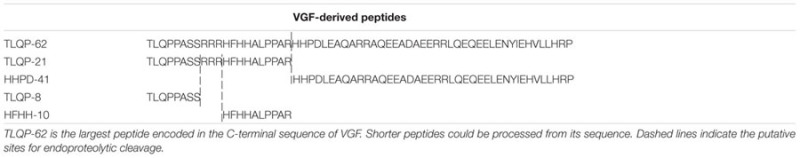

### TLQP-21 Binding Analysis in CHO Cells

The addition of Tyr-Ala (YA) amino acidic residues to TLQP-21 resulted in a peptide (YATL-23) with the same *in vitro* biological activity of TLQP-21 in CHO cells (data not shown).

**FIGURE 3:**
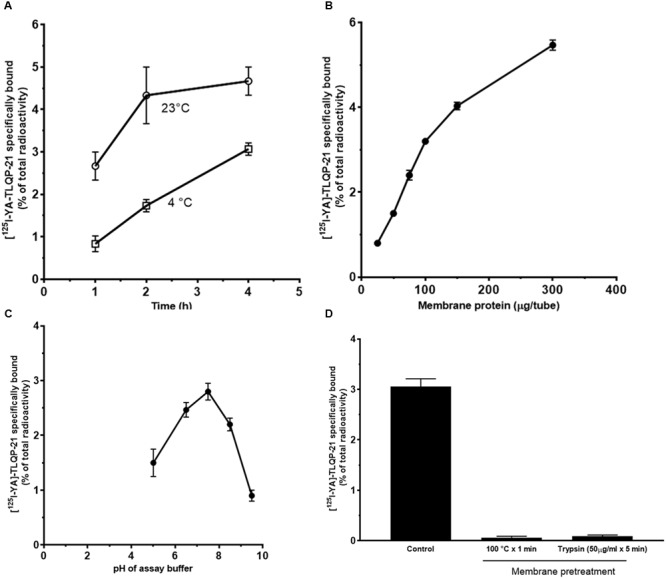
**Specific binding of [^125^I]-YATL-23 to CHO cell membranes as a function of the length of assay incubation at 23°C or 4°C (A)**, of the amount of membrane protein **(B)**, of pH of the incubation medium **(C)** and after membrane treatment at 100°C or with trypsin **(D)**. Values are means ± SEM of three independent experiments.

Given the evidence of intracellular Ca^2+^ mobilization induced by TLQP-21 in CHO cells, we investigated the presence of specific TLQP-21 binding sites on CHO cell membranes by radioreceptor assay using [^125^I]-YATL-23 as a ligand. The specific binding of [^125^I]-YATL-23 to cell membranes varied with both incubation time and temperature. Specific binding increased with the duration of incubation and was greater at 23°C when compared to 4°C after reaching equilibrium at 4 h (**Figure 3A**). A study of specific binding as a function of membrane protein concentration indicated that the binding was proportional to protein content up to at least 100 μg/tube (**Figure 3B**). Specific binding of [^125^I]-YATL-23 to cell membranes occurred over a relatively wide range of pH. The maximal binding was observed at pH 7.4 and declined to half-maximal value at pH 5.0 and 9.0 (**Figure 3C**). Therefore, all subsequent incubations were carried out at pH 7.4 for 4 h at 23°C with 100 μg membrane protein. Brief exposure of CHO cell membranes to high temperature (100°C × 1 min) or enzymes that disrupt protein structure such as trypsin (50 μg/ml × 5 min) caused the loss of the specific binding of [^125^I]-YATL-23, suggesting that protein integrity is functionally important in the binding site (**Figure 3D**).

**FIGURE 4:**
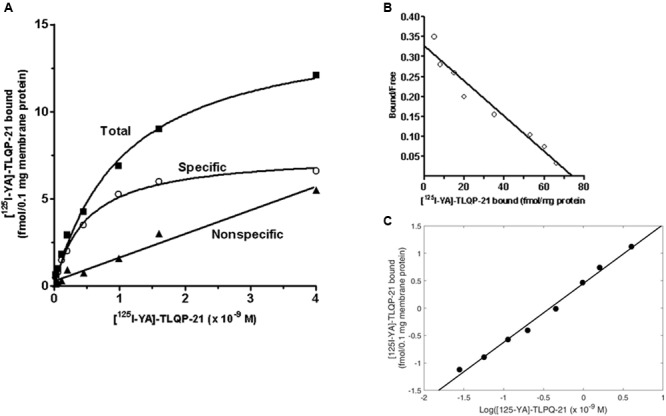
**Representative saturation isotherms, Scatchard and Hill plot of [^125^I]-YATL-23 binding to CHO cell membranes. (A)** Experiments were performed by incubating a fixed amount of membrane proteins with increasing concentrations of radiolabelled YATL-23, either alone (total binding) or together 1 μM unlabelled YATL-23 (nonspecific binding), respectively. Specific binding values were obtained subtracting nonspecific binding from total binding. **(B)** The saturation curve of specific binding was analyzed by Scatchard analysis. Values of *K*_d_ = 0.55 ± 0.05 × 10^-9^ M and *B*_max =_ 81.7 ± 3.9 fmol/mg protein were calculated using data obtained in three independent experiments. **(C)** Hill plot of the same data. The calculated Hill slope of 1.07 ± 0.1 is indicative of a single class of binding sites.

Experiments using various concentrations of [^125^I]-YATL-23 indicated the presence of a saturable and specific binding associated with minor low non-specific binding (**Figure 4A**). Scatchard transformation of the specific binding data (**Figure 4B**) yielded a linear Scatchard plot with Hill slope close to 1 (**Figure 4C**), indicating the existence of a single class of binding sites. The calculated *K*_d_ and *B*_max_ values (means ± SEM of three independent experiments) were 0.55 ± 0.05 × 10^-9^ M and 81.7 ± 3.9 fmol/mg protein, respectively, and the Hill slope was 1.07 ± 0.1.

**FIGURE 5:**
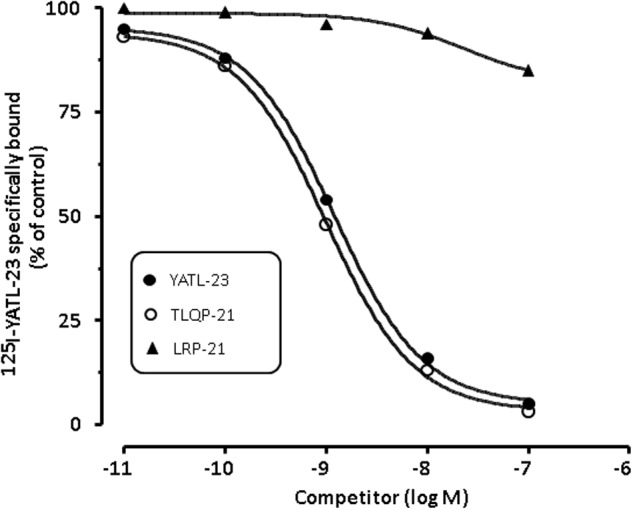
**Representative competition binding curves for [^125^I]-YATL-23 to CHO cell membranes by the indicated competitors.** Binding is expressed as percentage of control (specific binding in the absence of unlabelled competitor). Values are the means ± SEM of measurements obtained in six different wells for each experiment.

Unlabelled YATL-23 and TLQP-21 competed in a concentration-dependent manner with [^125^I]-YATL-23 for binding sites in CHO membranes (**Figure 5**). The IC_50_ values, calculated according a one-site binding model (means ± SEM of three independent experiments), were 1.2 ± 0.06 × 10^-9^ M for YATL-23 and 0.98 ± 0.06 × 10^-9^ M for TLQP-21. The results of these competition binding studies also revealed that the binding of [^125^I]-YATL-23 to CHO membranes was specific and was not inhibited by LRPS-21, the scrambled peptide made using the same amino acidic residues of TLQP-21 (**Figure 5**). These findings provide evidence that CHO cells contain significant amounts of TLQP-21 binding sites showing typical features of ligand-receptor interaction.

### Intracellular Transduction Mechanisms Activated by TLQP-21

**FIGURE 6:**
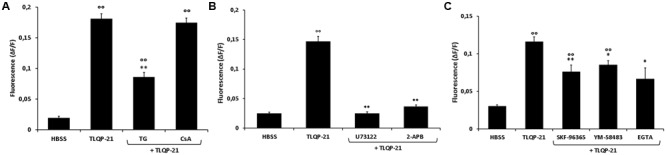
**Modulation of intracellular Ca^2+^ levels by TLQP-21 in the presence of different inhibitors in CHO cells.** Cells were loaded with FLUO-4 NW and treated with different inhibitors before the stimulation with 1 μM TLQP-21. **(A)** CsA (2 μM, 15 min), a mitochondrial PTP inhibitor, did not affect TLQP-21 Ca^2+^ stimulation as did TG (2 μM, 20 min), an inhibitor of the ER Ca^2+^-ATPase. ANOVA: *F* = 23.2; DF = 46. **(B)** The PLC-inhibitor U73122 (10 μM, 10 min) and the IP_3_R antagonist 2-APB (75 μM, 15 min) strongly reduced TLQP-21 stimulation of intracellular Ca^2+^ mobilization. ANOVA: *F* = 154.4; DF = 35. **(C)** SKF-96365 (10 μM, 20 min), YM-58483 (10 μM, 20 min) and EGTA (1 mM, 30 min), inhibitors of the SOCs pathway, affect significantly TLQP-21 effect. ANOVA: *F* = 20.4; DF = 49. Graphs show the means ± SEM of measurements obtained in at least six different wells for each experiment. All experiments have been repeated at least three times. ^∘∘^*p* < 0.01 vs. HBSS; ^∗^*p* < 0.05 and ^∗∗^*p* < 0.01 vs. TLQP-21.

It is known that Ca^2+^ stores, such as ER and mitochondria, dynamically participate in generation of cytoplasmic Ca^2+^ signals (Verkhratsky and Petersen, 1998). We therefore studied the possible implication of these intracellular organelles in the increase of intracellular Ca^2+^ levels induced by TLQP-21. The involvement of the ER was investigated using TG, which inhibits the Ca^2+^-ATPase pump responsible for sequestering Ca^2+^ in the ER and depletes the store by irreversibly preventing its refilling. As shown in **Figure 6A**, TG reduced the TLQP-21-mediated increase of intracellular Ca^2+^, causing a 56% reduction after 20 min preincubation time. To rule out the possibility that Ca^2+^ release from the mitochondria could also be involved, we evaluated the effect of TLQP-21 in the presence of CsA, an inhibitor of the mitochondrial PTP (Chernyak, 1997). The incubation of CHO cells with 2 μM CsA did not modify the basal levels of intracellular Ca^2+^ and a subsequent stimulation with 1 μM TLQP-21 induced a significant increase in intracellular Ca^2+^ levels (**Figure 6A**). These results indicate that TLQP-21 stimulated Ca^2+^ release primarily from the ER store, whereas release of Ca^2+^ from the mitochondria appeared not involved in this mechanism of action.

In many cellular systems, PLC activation and subsequent IP_3_ production is the transduction pathway regulating the release of Ca^2+^ from ER. U73122 is an aminosteroid that is reported to act as a specific inhibitor of PLC (Bleasdale et al., 1990) and it is widely used as a quick test for the involvement of PLC in a signaling pathway. To test whether TLQP-21 induces Ca^2+^ mobilization through a PLC-dependent mechanism, CHO cells were incubated for 10 min with 10μM U73122. Results demonstrate that U73122 induced a dramatic reduction, about 82%, of TLQP-21 stimulation of intracellular Ca^2+^ mobilization (**Figure 6B**). As a further confirmation, CHO cells were pre-incubated for 15 min with 75 μM 2-APB, which rapidly inhibits IP_3_R-mediated Ca^2+^ release. Treatment with 2-APB strongly affect TLQP-21 activity, causing a decrement of intracellular Ca^2+^ mobilization of about 79% (**Figure 6B**). As previously reported, depletion of Ca^2+^ from the ER causes the activation of stromal interaction molecule (STIM) proteins that, translocating into junctions formed between the ER and the plasma membrane (PM), activate the highly calcium-selective Orai channels to homeostatically balance intracellular calcium (Soboloff et al., 2012). To assess the role of this pathway, CHO cells were pretreated with a STIM-mediated Ca^2+^ inhibitor (SKF-96365), an inhibitor of Orai channels (YM-58483) or an extracellular Ca^2+^ chelator (EGTA) before stimulation with 1 μM TLQP-21. We observed a statistically significant decrement in TLQP-21-mediated intracellular Ca^2+^ mobilization (**Figure 6C**), of about 36, 27, and 45% after treatment with SKF-96365, YM-58483, and EGTA, respectively.

**FIGURE 7:**
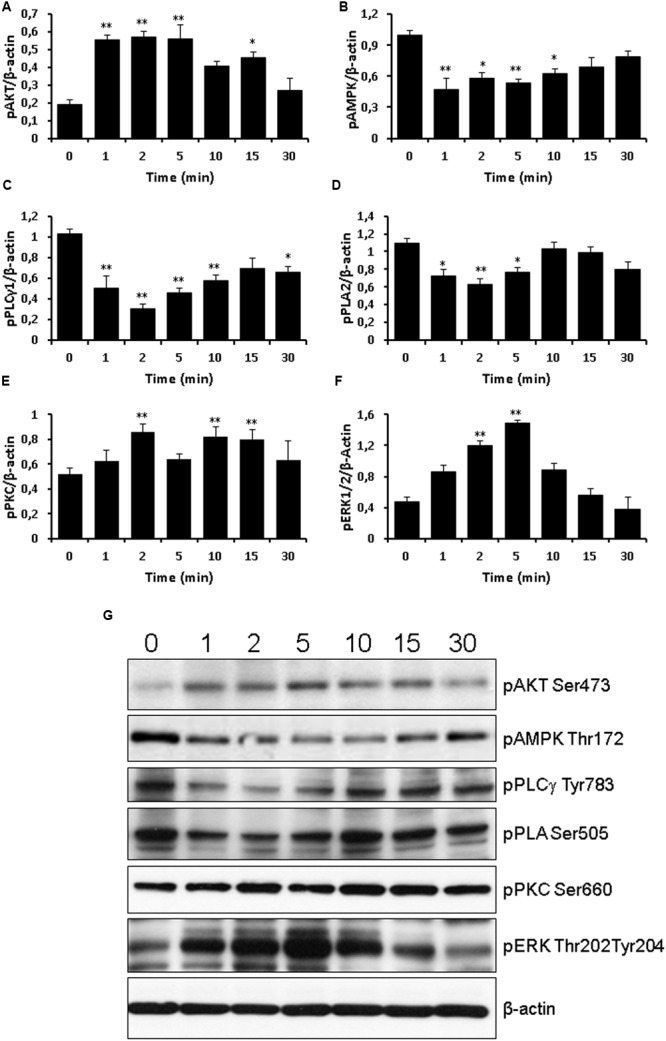
**Western blot analysis of signaling phospho-activated proteins.** Time course of 10 μM TLQP-21 treatment from 1 min up to 30 min. Equal amounts of cell lysates were processed for Western blot analysis, using specific antibodies for the phosphorylated (active) forms of signaling molecules. Abbreviations: pAMPK = phospho-AMPK (Thr172); pAKT = phospho-AKT (Ser473); pERK1/2 = phospho-ERK1/2 (Thr202/Tyr204); pPKC = phospho-PKC (Ser660); pPLA2 = phospho-PLA2 (Ser505); and pPLCγ1 = phospho-PLCγ1 (Tyr783). To ensure equal loading, blots were also probed with anti-β-actin. **(A–F)** Results shown are means ± SEM of at least five measurements obtained in five independent experiments. ^∗^*p* < 0.05 and ^∗∗^*p* < 0.01 vs. time 0. **(G)** representative images of the blots.

Next, we measured the effects of TLQP-21 treatment on phosphorylation of intracellular signaling effectors. After 1 h of serum starvation, cells were exposed to 10 μM TLQP-21 for 0–30 min (**Figure 7**). The 10 μM concentration was chosen because it elicited a stimulation near to maximal on intracellular Ca^2+^ mobilization (**Figure 1**). TLQP-21 induced a prompt increase of phospho-AKT (1–5 min) (**Figure 7A**) that remained significantly higher than basal until 15 min after stimulation (**Figure 7A**). A rapid and transient dephosphorylation of phospho-AMPK and phospho-PLCγ1 occurred between 1 and 10 min after stimulation with TLQP-21(**Figures 7B,C**). A similar trend was seen also for phospho-PLA2, but dephosphorylation reached statistical significance only at 2 min (**Figure 7D**). Interestingly, PKC phosphorylation increased after 2 min and lasted until 15 min from stimulation with TLQP-21 (**Figure 7D**). ERK1/2 phosphorylation increased significantly at 2 min, reached a peak at 5 min and thereafter returned to basal (**Figure 7F**). No effects were induced on phospho-JNK levels (data not shown). A possible limitation of these determinations is that sample loading was normalized using the corresponding β-actin levels and not the levels of the unphosphorilated protein.

## Discussion

The molecular mechanisms of TLQP-21 action at the cellular level are still an object of debate, although this peptide could represent an important target to counteract human disorders, including obesity and diabetes. Our results demonstrate that TLQP-21 is a robust extracellular signal capable of promoting a Ca^2+^-mediated transduction signal in CHO cells. TLQP-21 is one of many bioactive products derived from post-translational cleavage of the C-terminal region of VGF, a propeptide that could be involved in psychiatric, neurologic and metabolic disorders (Carrette et al., 2003; Ruetschi et al., 2005; Bartolomucci et al., 2011; Busse et al., 2012). Although TLQP-21 has been implicated in the regulation of energy balance, nociception, gastric function, and several other physiologic functions, its binding site and mechanisms of action remain largely unknown (Bartolomucci et al., 2006; Rizzi et al., 2008; Chen et al., 2013). We have investigated the ability of some VGF-derived peptides to induce intracellular Ca^2+^ mobilization in CHO cells. Our results show that: (i) TLQP-21 stimulates a sharp increase in free intracellular Ca^2+^ levels; (ii) on this pathway it is more active than other VGF-derived peptides; and (iii) its C-terminal fragment, that corresponds to HFHH-10 peptide, is the region primarily involved in the process of stimulating intracellular Ca^2+^ mobilization. These results are consistent with those indicating that the “hot spots” for TLQP-21 activity are localized in its C-terminal region (Cero et al., 2014). The N-terminal region of the peptide appears more tolerant to modifications, since the introduction of Tyr-Ala (YA) amino acidic residues (YATL-23) did not change the activity on intracellular Ca^2+^ mobilization and the ability of the peptide to bind the receptor. However, the N-terminal region of TLQP-21, corresponding to the sequence of TLQP-8, is apparently devoid of any activity on intracellular Ca^2+^ mobilization. Interestingly, molecular dynamics simulations have revealed that TLQP-21 is characterized by a highly flexible nature that fluctuates between many conformations, spanning from a long helical to a more compact form (Chakraborty et al., 2015). Another factor adding complexity to the interpretation of the ability of VGF-derived fragments to activate their receptor is the demonstration that TLQP-21 undergoes a folding-upon-binding transition upon receptor binding (Cero et al., 2014). Further studies are needed to understand whether HFHH-10 satisfies the random coil to helix transitions needed for activating G-protein coupled receptors (Shoichet and Kobilka, 2012).

The results that we have obtained in this research clearly demonstrated that TLQP-21 interacts with a single class of binding site expressed on CHO cell membranes, further confirming those observations that we have previously reported using Atomic Force Microscopy (Cassina et al., 2013). It is interesting that the membranes of CHO cells contain significant amounts of TLQP-21 binding sites and this cell line can be a useful *in vitro* model to study TLQP-21 mechanisms of action. In our setting, TLQP-21 efficiently stimulated intracellular calcium levels in CHO cells without the need of ATP priming previously reported (Hannedouche et al., 2013). Ca^2+^ is a highly versatile second messenger involved in a variety of intracellular signaling pathways, including gene regulation, proliferation and cell death. Repeated exposure to TLQP-21 resulted in a reduced response, probably indicating a decrease in number of receptors available or a decreased activity of intracellular signaling pathways involved. Interestingly, TLQP-21 induced a specific desensitization to subsequent TLQP-21 treatments, but not to ATP, and these data are consistent with those reported in rat primary macrophages and cerebellar granule cells (Severini et al., 2008; Chen et al., 2013).

We have also shown that TLQP-21 stimulation induced an increase of cytoplasmic Ca^2+^ levels that was determined by release of Ca^2+^ from intracellular stores. In particular, our studies indicate that in CHO cells Ca^2+^ is released mainly from the ER stores, as demonstrated by the treatment with TG, a Ca^2+^-ATPase pump inhibitor that depletes the ER store by preventing its refilling (Thastrup et al., 1989), whereas Ca^2+^ release from the mitochondria did not appear involved. Our results also indicate that Ca^2+^ mobilization stimulated by TLQP-21 is triggered by the activation of PLC, since pretreatment of the cells with U73122, a known PLC inhibitor, induced a significant reduction of Ca^2+^ mobilization. Western blotting determinations indicated that TLQP-21 up-regulated the phosphorylation levels of ERK1/2, PKC and AKT in CHO cells. These kinases are activated by the dual phosphorylation of neighboring threonine and tyrosine residues in response to extracellular stimuli (Nadeau and Landry, 2007) Furthermore, our results show that TLQP-21 induced a rapid and transient dephosphorylation of AMPK, PLCγ1, and PLA2, whereas the phosphorylation state of JNK was not altered. The intricate balance of phosphorylation by kinases and dephosphorylation by phosphatases is essential for maintaining signal transduction networks in cells (Barford et al., 1998). The rapid increase in the mobilization of intracellular Ca^2+^ is at least in part dependent on the PLCγ family of proteins, including PLCγ1 and PLCγ2 (Clapham, 2007). It is possible that the dephosphorylation of PLCγ1 that we have observed could be involved in the mechanisms by which TLQP-21 induced a clear desensitization to subsequent TLQP-21 treatments. These results suggest that the receptor stimulated by TLQP-21 should belong to the family of the G_q_-coupled receptors. It is widely accepted that the IP_3_R on the ER are an essential link between PLC activation and initiation of Ca^2+^ release from the ER. We have shown that pretreating the CHO cells with 2-APB, an IP_3_Rs antagonist, the release of Ca^2+^ induced by TLQP-21 was heavily reduced, further confirming that the binding of TLQP-21 with its receptor activates the PLC pathway. Reportedly, rapid Ca^2+^ depletion from the ER activates slower Ca^2+^ entry from outside the cell (Hewavitharana et al., 2007). STIM and Orai proteins are required for the store-operated Ca^2+^ entry process (Liou et al., 2005; Roos et al., 2005). Consistently, we have found an inhibition of TLQP-21-mediated Ca^2+^ release following treatment with SKF-96365 and YM-58483, two specific STIM- and Orai-inhibitors, respectively. PLC activation by TLQP-21 is followed by an increase of PKC phosphorylation and, afterwards, ERK1/2 phosphorylation.

Given the previous identification of two putative receptors for TLQP-21, the C3aR1 in CHO and RAW264.7 cells, and the gC1qR in rat brain membranes, primary microglia, macrophages, and DRG neurons (Chen et al., 2013; Hannedouche et al., 2013), we have attempted its pharmacological characterization in CHO cells. We have also previously demonstrated the existence of a high-affinity binding site for TLQP-21 in white and brown adipocyte membranes, and adrenals, whose exact nature remains still unknown (Possenti et al., 2012). CHO cells could be a useful in vitro model to study the interaction of VGF-derived peptides with a putative specific binding site, but the possibility that different binding sites could be expressed by other cell systems cannot be ruled out. Understanding the mechanism of action of TLQP-21 could be of great relevance since this neuropeptide is purported to play important roles in the central nervous system and peripheral tissues.

## Conclusion

Our results suggest that TLQP-21 effects in CHO cells could be mediated by a G_q_-coupled receptor that quickly activates PLCβ, that produces DAG and IP_3_ as second messengers. DAG activates PKC that, in turn, stimulates ERK1/2 phosphorylation. On the other hand, by binding IP_3_R on the ER, IP_3_ stimulates a sharp increase of intracellular Ca^2+^ levels and the subsequent Ca^2+^ entry from outside the cell through STIM-Orai interaction (see Graphical abstract). The CHO cell line can be proposed as a useful model for developing new synthetic agonists and antagonists of TLQP-21 receptor, which could help a better understanding of the physiologic and pathological role of VGF-derived peptides.

## Ethics Statement

The study was made *in vitro* using cell lines only. No experiments were performed on animals or involved human beings. Approval from the local Ethic Committee or other regulatory agencies was not required.

## Author Contributions

AT, RP, VL, and RO supervised the entire project, designed research, and wrote the paper. GB and IR conceived and designed the experiments, interpreted and analyzed data, supervised all the experimental procedure. LM, LR, EB, PP, CG, GM, J-AF, PV, AB, and JM conceived and designed the experiments, performed research, interpreted, and analyzed data. AT, RO, and VL analyzed data and critically revised the manuscript.

## Conflict of Interest Statement

The authors declare that the research was conducted in the absence of any commercial or financial relationships that could be construed as a potential conflict of interest. The reviewer JH and handling Editor declared their shared affiliation, and the handling Editor states that the process nevertheless met the standards of a fair and objective review.
